# Novel *FOXL2* mutations in two Chinese families with blepharophimosis-ptosis-epicanthus inversus syndrome

**DOI:** 10.1186/s12881-015-0217-7

**Published:** 2015-09-01

**Authors:** Min Xue, Jie Zheng, Qing Zhou, J. Fielding Hejtmancik, Yuan Wang, Shouling Li

**Affiliations:** Department of Ophthalmology, the First Affiliated Hospital of Anhui Medical University, Hefei, China; National MOE Key Laboratory of Gene Resource Utilization for Important Genetic Disease, Anhui Key Laboratory of Genetic Research, Hefei, China; Ophthalmic Genetics and Visual Function Branch, National Eye Institute, National Institutes of Health, Bethesda, MD USA; Department of Ophthalmology, Anhui NO.2 Provincial people’s hospital, Hefei, China

## Abstract

**Background:**

Blepharophimosis-ptosis-epicanthus inversus syndrome (BPES) is a rare autosomal dominant disease. Mutations in the forkhead box L2 (*FOXL2)* gene cause two types of BPES distinguished by the presence (type I) and absence (type II) of premature ovarian failure (POF). The purpose of this study was to identify possible mutations in *FOXL2* in two Chinese families with BPES.

**Methods:**

Two large autosomal dominant Chinese BPES families were enrolled in this study. Genomic DNA was obtained from the leukocytes in peripheral venous blood. Four overlapping sets of primers were used to amplify the entire coding region and nearby intron sequences of the *FOXL2* gene for mutations detection using polymerase chain reaction (PCR) and sequencing analyses. The sequencing results were analyzed using DNAstar software.

**Results:**

All patients of the two families demonstrated typical features of BPES type II, including small palpebral fissures, ptosis, telecanthus, and epicanthus inversus without female infertility (POF). A novel *FOXL2* heterozygous indel mutation c.675_690delinsT, including a 16-bp deletion and a 1-bp(T) insertion (p.Ala226_Ala230del), which would result in deletion of 5 alanine residues of a poly-alanine (poly-Ala) tract in the protein, was identified in all affected members of family A. A novel heterozygous missense mutation (c.223C > T, p.Leu75Phe) was identified in family B.

**Conclusions:**

Two novel *FOXL2* mutations were identified in Chinese families with BPES. Our results expand the spectrum of *FOXL2* mutations and provide additional structure-function insights into the FOXL2 protein.

## Background

Blepharophimosis-ptosis-epicanthus inversus syndrome (BPES; OMIM# 110100) is an autosomal dominant developmental disorder characterized by a malformation of the eyelids. It has a prevalence of approximately 1 in 50,000 [1]. Depending on the presence or absence of premature ovarian failure (POF), BPES has been divided into two subsets: type I is characterized by eyelid malformation and female infertility through POF, and type II is characterized by eyelid malformation [2]. Clinically, patients with BPES experience a combination of congenital eyelid anomalies characterized by a reduction of the horizontal fissure length, ptosis, telecanthus and epicanthus inversus (that is, below the inner canthus). The BPES gene was mapped to the 3q23 chromosomal region [3–7], and mutations in the forkhead box L2 (*FOXL2*) gene (OMIM# 605597) were subsequently identified and associated with both types of BPES [8].

Human FOXL2 is a member of the winged helix/forkhead transcription factor family. Expression studies have revealed that the FOXL2 protein is present in the mesenchyme of developing eyelids, in fetal and adult granulosa cells of the ovary, and in the embryonic and adult gonadotropic cells of the anterior pituitary [8–12], suggesting that this protein might play a key role in regulating the early development of the eyelids and ovaries and maintaining the female gonads in vertebrate species [8, 10].

Of all genetic defects identified in BPES, intragenic mutations represent the largest group (71 %) [13]. Deletions encompassing *FOXL2* and located outside its transcription unit represent 12 % and 5 % of molecular defects respectively [14]. The largest group (44 %) of unique *FOXL2* mutations contains frameshift mutations. Following are the in-frame changes (33 %, of which poly-Ala expansions represent the largest group), the nonsense mutations (12 %) and finally the missense mutations (11 %) [13]. Several genotype-phenotype correlations emerged after the identification of the *FOXL2* mutations. Some authors have found that mutations resulting in a predicted truncated protein before the poly-Ala tract are associated with BPES type I, whereas mutations resulting in an extended protein might lead to BPES type II, but no clear genotype-phenotype correlations have been confirmed between mutations and BPES types. This is because the genetic and clinical heterogeneity are found in and between families with BPES [15, 16].

Here, we report two novel *FOXL2* heterozygous mutations identified in Chinese families with BPES. Both a c.675_690delinsT (p.Ala226_Ala230del) indel mutation and a c.223C > T (p.Leu75Phe) missense mutation are associated with BPES type II.

## Methods

### Patients

**Fig. 1:**
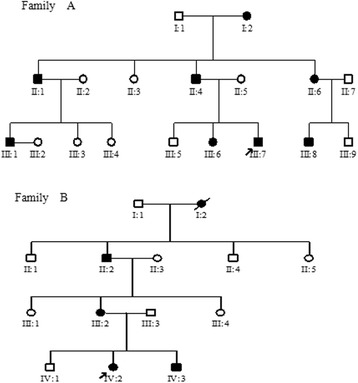
Pedigrees of two Chinese families

The two large Chinese families with BPES were ascertained through the First Affiliated Hospital of Anhui Medical University (Fig. 1). A total of 31 individuals, including 12 affected individuals, were recruited in this study. An ophthalmologist performed detailed examinations of the patients, and the following criteria were used to accept a diagnosis of BPES [17]: blepharophimosis, ptosis, epicanthus inversus, and telecanthus. POF was defined as the cessation of ovarian function under the age of 40 years and characterized by amenorrhoea, hypoestrogenism and elevated serum gonadotrophin concentrations. Besides, in order to distinguish the type of BPES, the clinical data of female patients were particularly concerned about (Table 1). The proband of family A (III:7), a boy, was 10 years old and got the pathogenic gene from his father. The girl proband of family B (IV:2) was diagnosed as BPES when she was 8. At the time of her birth, her mother was a 28-year-old BPES patient. Both of the young probands showed typical features of BPES, including small palpebral fissure, ptosis of the eyelids, epicanthus inversus, and telecanthus.

**Table 1:** The clinical details of female patients

Patient	Age	Menarche age	Menopause age	Pregnancies No.	The last pregnancy age
FamilyA					
I:2	63	13	52	4	30
II:6	34	14	Not happen	2	27
III:6	11	Not happen	--	--	--
FamilyB					
I:2	--	Unknow	About 50	4	34
III:2	36	13	Not happen	3	31
IV:2	8	Not happen	--	--	--

Informed consent was obtained from all participants or their guardians for research according to the tenets of the Declaration of Helsinki and Guidance of Sample Collection of Human Genetic Diseases through the Ministry of Public Health of China.

### Mutational analysis

Blood samples were collected from all the individuals of both Chinese families, and genomic DNA was extracted from the leukocytes of peripheral venous blood using the phenol-chloroform method. For mutation analysis, the PCR amplification of the genomic fragments encompassing *FOXL2* coding regions was performed using four overlapping sets of primers [8].

PCR was performed with 100 ng genomic DNA, 25 μl 2 × GC-rich buffer, 8 μl of a dNTP mixture (2.5 mmol/L), 1 U LA Taq (Takara Biotechnology (Dalian) Co., Ltd), 2 μl (10 μmol/L) of each primer, and ddH_2_o to a final volume of 50 μl. The PCR amplification was performed initially at 94 °C for 5 min, followed by 35 cycles at 94 °C for 60 s, at 60 °C or 63 °C for 50 s, and 72 °C for 60 s, with a final elongation step at 72 °C for 10 min.

**Fig. 2:**
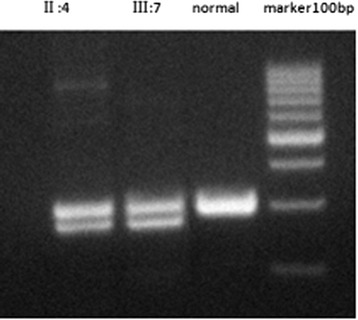
The PCR products amplified by primer 4 in the Family **a**. Patients’ PCR products revealed two fragments of 304 and 289 bp using 6 % agarose gel electrophoresis. Normal individual contained a single fragment of 304 bp. The right lane is DNA marker (100bp)

Analysis using agarose gel electrophoresis revealed two fragments in the PCR products amplified by primer 4 in the Family A patients, while those of unaffected individuals contained a single fragment (Fig. 2). Because these PCR products could not be adequately sequenced directly, the PCR fragments were purified and cloned into the PMD18-T vector (Takara Biotechnology (Dalian) Co., Ltd). The constructs were subsequently transformed into competent DH5α Escherichia coli. The plasmids were extracted from the positive clones. The cloned fragments of two types were identified by 6 % agarose gel electrophoresis after PCR using the primers mentioned above. The plasmids were subsequently sequenced on an ABI-3730 DNA Analyzer (Takara Biotechnology (Dalian) Co., Ltd) using BcaBEST sequencing primers RV-M: 5’-GAGCGGATAACAATTTCACACAGG-3’ and M13-47: 5’-CGCCAGGGTTTTCCCAGTCACGAC-3’.

All other PCR products were analyzed through direct sequencing using the same sequencing primers as in the PCR amplification. The sequencing results were analyzed by the Seqman sequence assembly program within DNAstar software.

Mutation analysis of the entire *FOXL2* gene was performed in all living affected members of two families. Both variations detected in *FOXL2* gene were further evaluated in the family unaffected members as well as in 100 normal controls.

## Results

**Fig. 3:**
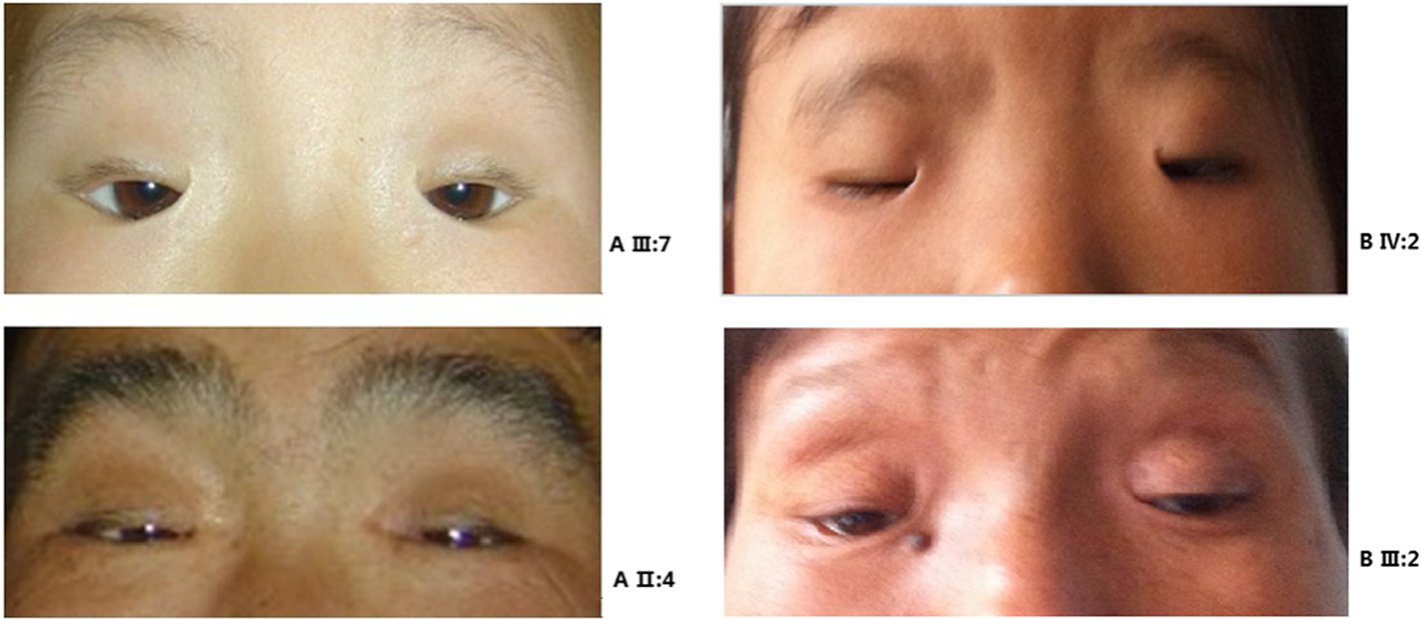
Pictures representing the ocular defects of BPES patients from two Chinese families. Patients experience a combination of congenital eyelid anomalies: small palpebral fissures, ptosis, telecanthus, and epicanthus inversus. The left is Family (**a**) and the right is Family (**b**)

All patients in two families demonstrated the typical features of BPES, including small palpebral fissures, ptosis, telecanthus and epicanthus inversus. None of the patients exhibited microcephaly, intellectual disability or other malformations (Fig. 3), and all adult female patients had offspring (Fig. 1). From the clinical date (Table 1), female patients have no signs of POF, although hormone levels data are not available.

**Fig. 4:**
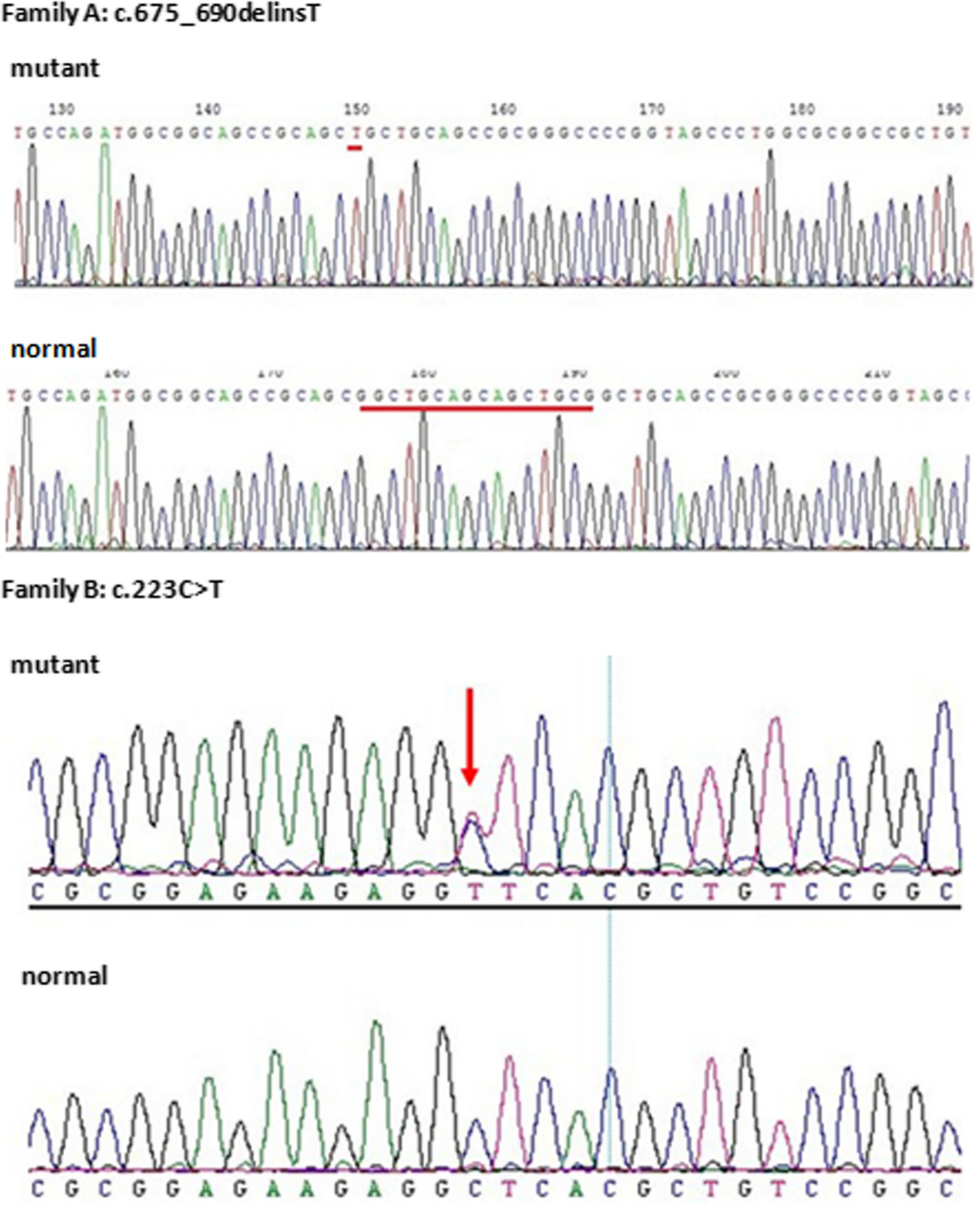
Sequencing results of the two novel mutations in *FOXL2* (the heterozygous mutation c.675_690delinsT in family **a** and the missense mutation (c.223C > T) in family **b**). The mutant alleles identified in the present study are compared with the normal alleles. The red marks indicate the position of mutations

The PCR products obtained from all the patients of family A included two fragments (304 and 289 bp) on agarose gel electrophoresis. The sequence of the cloned PCR products obtained from these patients revealed a novel heterozygous indel mutation, c.675_690delinsT, a 16-bp deletion and a 1-bp (T) insertion (Fig. 4). This mutation results in a p.Ala226_Ala230del, a deletion of 5 alanine residues of poly-Ala tract in the protein.

A novel heterozygous missense mutation (c.223C > T, p.Leu75Phe) was detected in the family B (Fig. 4), which is in the forkhead domain of the *FOXL2*. This change was assessed to be deleterious (PROVEAN score = −3.185) by analysis with the PROVEAN online tool (http://provean.jcvi.org/index.php), to be not tolerated by SIFT (http://sift.bii.a-star.edu.sg/index.html), to be probably damaging by PolyPhen-2 with a score of 0.999 (http://genetics.bwh.harvard.edu/pph2/index.shtml), and to be disease causing by mutation taster (http://www.mutationtaster.org/) (Table 2).

**Table 2:** *FOXL2* mutation (c.223C > T) results from pathogenicity prediction software

	PROVEAN	SIFT	PolyPhen-2	Mutation Taster
Score	−3.185	0.00	0.999	0.999
Prediction Result	deleterious	Affect protein function	Probably damaging	disease causing

Testing of each mutation showed presence in all affected family members respectively in a heterozygous state, and absence in the unaffected members and 100 normal controls.

## Discussion

FOXL2 is a forkhead transcription factor, containing a forkhead domain for DNA binding and a poly-Ala tract [8, 18]. It is encoded by a 2.7-kb DNA sequence with only one exon that is translated into a protein of 376 amino acids. FOXL2 is highly expressed in developing eyelids [10, 19], and in the fetal and adult granulosa cells of the ovary [9–12]. Mutations in *FOXL2* are responsible for BPES both type I and type II [8]. To date, more than 200 mutations in *FOXL2* have been associated with BPES in different populations [14, 17]. Previous studies have shown the existence of two mutational hotspots: 30 % of *FOXL2* mutations lead to poly-Ala expansions, and 13 % are a novel out-of-frame duplication [15].

In our study, none of the female patients with amenorrhoea were mentioned before the age of 40 in clinical inquiry. The I: 2 female patient of family A was 63 years old. Her menopause was at 52 years old which was much older than fourty. Moreover, when she was thirty, she gave birth to a daughter (II: 6). 34-year-old II: 6 was also a BPES patient with regular menstrual cycle, and was two boys’ mother. The female fertility situation of family B was the same as family A. The affected adult females in family B did not have fertility problems, but with regular menstrual cycle. All patients in these two families exhibited the typical eyelid malformation of BPES, small palpebral fissures, ptosis, telecanthus and epicanthus inversus, but without female infertility (POF), suggesting that the phenotypes of the patients in two families were BPES type II.

Affected individuals in family A showed an indel mutation c.675_690delinsT, consisting of a 16-bp deletion and 1-bp (T) insertion, which is predicted to remove 5 out of 14 alanine residues (p.Ala226_Ala230del) from the poly-Ala tract located downstream of the winged helix/forkhead domain of the FOXL2 protein. The number of alanine residues is strictly conserved among the human, mouse, rat, and goat, suggesting the existence of strong functional or structural constraints [9]. The exact role of the poly-Ala tract in FOXL2 is unknown, but the mechanism for the molecular pathogenesis of the poly-Ala expansions in BPES has been suggested to be a result of cytoplasmic aggregation of the FOXL2 protein as well as inclusion in nuclear aggregates [16, 20–22], most often leading to BPES type II [15, 23]. However, this correlation is subject to both intra and inter familial phenotypic variations [8, 15, 16, 23, 24]. In this study, the mutation c.675_690delinsT causes removal of 5 out of 14 alanine residues (Ala-5), leading to a truncation at the poly-Ala tract, showing that either contraction or expansion of the poly-Ala tract can result in BPES type II. This is probably because strong structural or physico-chemical constraints are imposed to the length of the polyAla tract: expansion induces cytoplasmic retention as well as cytoplasmic and nuclear aggregation. Contraction would also be deleterious in the context as the protein might adopt an incorrect conformation leading to intranuclear aggregation [22]. It is the first report that a partial deletion of the poly-Ala tract in *FOXL2* is associated with BPES type II, but the other poly-Ala tract partial deletion (p.Ala221_Ala230del) has been described in one POF patient with no eye defects [25]. Our data further support that there are no clear genotype-phenotype correlations between mutations and BPES types because the genetic and clinical heterogeneity are found in and between families with BPES.

Family B showed the c.223C > T (p.Leu75Phe) missense mutation, which lies within the forkhead domain, seems likely to interfere with DNA binding, although it doesn’t lie within a specifically identified DNA binding site. Most missense mutations identified in *FOXL2* occur within the forkhead domain [15]. Missense changes have been suggested to act as null allele leading to BPES phenotype due to haploinsufficiency [16] or dominant-negative effect [26]. Previous studies indicated that missense mutations inside or outside the forkhead domain could determine the expressivity of BPES. Mutations inside the forkhead domain might produce a more severe phenotype, while mutations outside it might produce a mild phenotype [27]. In our study, the BPES family carrying the p.Leu75Phe mutation has a typical clinical BPES phenotype, which further supports the possibility that the affected BPES individuals with missense mutation inside the forkhead domain might have a severe phenotype. The results obtained from pathogenicity prediction software suggest that this mutation may affect protein function. Taken together, these data testify that the functional alteration of FOXL2 transcription factor by the c. 223C > T substitution may be associated with this severe phenotype BPES.

## Conclusions

In conclusion, we found two novel mutations in the *FOXL2* gene in two large Chinese families with individuals affected with BPES type II. An indel mutation c.675_690delinsT (p.Ala226_Ala230del) occurs at the poly-Ala tract, predicted to remove 5 out of 14 alanine residues from the poly-Ala tract, leading to contraction of a highly conserved poly-Ala tract. It is the first report that a partial deletion of the poly-Ala tract in FOXL2 is associated with BPES type II. The second is a novel missense mutation c.223C > T (p.Leu75Phe) occurring within the forkhead domain, and probably interfering with DNA binding. These results expand our current understanding of the spectrum of *FOXL2* mutations, especially with regard to the poly-Ala tract.

## Abbreviations

BPES: Blepharophimosis-ptosis-epicanthus inversus syndrome
FOXL2: Forkhead box L2
POF: Premature ovarian failure
PCR: Polymerase chain reaction
Poly-Ala: Poly-alanine
